# Association Between Lung Parenchymal Attenuation in Computed Tomography and Airflow Limitation in Adults with Cystic Fibrosis

**DOI:** 10.3390/diagnostics15010107

**Published:** 2025-01-04

**Authors:** Lucía Esteban Baloira, Ester Zamarrón de Lucas, Carlos Carpio Segura, Macarena Lerín Baratas, María Fernández Velilla, María Isabel Torres Sánchez, Inmaculada Pinilla Fernández, Pablo Mariscal Aguilar, Rodolfo Álvarez-Sala Walther, Concepción Prados Sánchez

**Affiliations:** 1 Servicio de Neumología, Hospital Universitario La Paz, IdiPAZ, CIBERES, Universidad Autónoma de Madrid, 28046 Madrid, Spain; ester.zamarronde@salud.madrid.org (E.Z.d.L.); carlosjavier.carpio@salud.madrid.org (C.C.S.); macarena.lerin@salud.madrid.org (M.L.B.); pablo.mariscal@salud.madrid.org (P.M.A.); rodolfo.alvarezsala@salud.madrid.org (R.Á.-S.W.); mconcepcion.prados@salud.madrid.org (C.P.S.); 2 Servicio de Radiodiagnóstico, Hospital Universitario La Paz, IdiPAZ, Universidad Autónoma de Madrid, 28046 Madrid, Spain; mfvelillapena@salud.madrid.org (M.F.V.); isabel.torres@salud.madrid.org (M.I.T.S.); minmaculada.pinilla@salud.madrid.org (I.P.F.)

**Keywords:** cystic fibrosis, computed tomography, lung attenuation, airflow limitation

## Abstract

**Objectives**: To determine the association between airflow limitation and the quantification of lung attenuation in computed tomography (CT) in adult patients with cystic fibrosis (CF). **Methods**: A cross-sectional study in a single center between January 2013 and December 2018 in adult patients with stable CF. We collected clinical data and the results of spirometry and plethysmography. A chest CT at inspiration and expiration, using a specific software that automatically measured the lung attenuation, was performed. **Results**: In total, 73 patients (63% males) were included. The mean age was 31.6 ± 12.3 years and the FEV1 was 67.8 ± 25.9% pred. An airflow limitation was found in 63%, the mean residual volume was 159.9% pred, and air trapping was observed in 50 (87.7%) of the patients. The patients with airflow limitations showed a higher bulla index and a percentage of lung voxels in the range of emphysema. The FEV1 and the FEV1/FVC correlated with the percentage of the lungs at a high attenuation value (HAV), the range of emphysema, and the bulla index at inspiration, as well as the mean lung density at expiration and the inspiratory–expiratory variation of the mean lung density (MLDi-e). Finally, in the multivariate model, the MLDi-e and the HAV at inspiration were associated with airflow limitations. **Conclusions**: The measurements obtained from the automated quantification of lung parenchymal attenuation predicts airflow limitation in CF.

## 1. Introduction

Cystic fibrosis (CF) is the most frequent autosomal recessive multiorgan disease in Caucasians. It is caused by a defective cystic fibrosis transmembrane regulator (CFTR) protein, which generates thick secretions in the affected organs. In the airways and lung parenchyma, it is characterized by progressive inflammatory structural changes, as well as obstruction and air trapping that compromise the quality of life and the life expectancy of patients [1,2]. Pulmonary disease gradually worsens as individuals get older, although the pattern of change is unpredictable and varies greatly among subjects. Despite the important paradigm shift that the appearance of modulators has brought about in the last few years, the effect and, therefore, the progression of the disease may vary considerably from individual to individual as modulators may not be suitable or effective for all patients with cystic fibrosis [3,4].

Pulmonary function tests (PFTs) are strong predictors of mortality and morbidity [5]. However, although forced expiratory volume in one second (FEV1) is an established outcome in CF, it has poor sensitivity to evaluate peripheral airway disease. In this sense, multiple studies have validated the use of computed tomography (CT) findings as a prognostic value of CF, both due to its greater sensitivity than FEV1 in detecting the progression of lung disease and due to its correlation with exacerbations and the need for antibiotic treatment [6,7,8,9,10,11].

Most CF units usually performed chest CTs in a serial fashion, in combination with PFT, to improve the assessment of lung disease progression and to quantify the degrees of bronchiectasis, air trapping, and other characteristics related to structural changes due to CF lung disease, such as mucus plugging, bronchial wall thickening, or atelectasis [12,13]. However, there are other complementary attributes that it is possible to measure using CT, such as the lung parenchyma attenuation. A better characterization of the patients will allow for the detection of the progression of the disease with greater sensitivity.

Thus, in chronic obstructive pulmonary disease (COPD), another chronic respiratory disease that also causes airflow obstruction, a correlation has been shown between the analysis of the density of the lung parenchyma via CT and both the pathological alterations observed in tissue samples and an impaired lung function (airflow obstruction and diffusion capacity) [14,15,16,17]. To this end, several methods have been developed to objectively quantify pulmonary emphysema, mostly by measuring the mean lung density (MLD), the bulla index, the areas of the lung occupied by attenuation values lower than the predetermined thresholds, and a predetermined percentile of the pulmonary attenuation distribution curve. Regarding CF, most studies exploring lung density have been performed in children. Moreover, they primarily measured air trapping, a common finding in CF, indicated by areas of abnormally low lung attenuation on expiratory CT images [18,19,20]. However, there are other progressive morphological changes in the lung parenchyma observed in CF, such as bronchiectasis or mucus plugging, that can potentially affect the distribution of CT densities [21].

Due to most of the studies assessing lung attenuation in CF via CT being conducted with small groups of children [18,19,20], we hypothesize that the measures of pulmonary attenuation may adequately correlate with PFTs in adults. Therefore, the objectives of this study were (1) to associate lung function and lung attenuation and (2) to characterize lung CT attenuation; both in CF adult patients.

## 2. Materials and Methods

The ethical review board approved the study (PI-2531).

### 2.1. Study Population

We performed a cross-sectional, observational study of patients diagnosed with CF [22] who attended a tertiary hospital in Spain from January 2013 to December 2018. We included all the patients older than 18 years under follow-up in the CF unit who were stable, at least for the previous three months; following usual clinical practice; and underwent an automated quantitative analysis thorax CT scan. We excluded those patients with limitations that prevented the performance of a CT scan, for example, patients who were pregnant or were lung transplant recipients, as well as the those who refused this test.

We registered demographic data, history of exacerbations during the last year, sputum culture results, systemic inflammatory (C-reactive protein [CRP], erythrocyte sedimentation rate [ESR], fibrinogen) and nutritional biomarkers (albumin, proteins, proalbumin), spirometry and pletismography results.

Airflow limitation (AL) was defined as an FEV1/FVC ratio of <0.7.

### 2.2. CT Scanning Procedures and Quantitative Analysis

All the patients were scanned in stable clinical condition, on a multidetector CT scanner (16 rows, Somatom Emotion 16, Siemens Medical Solutions, Erlangen, Germany), following the same protocol. Both unenhanced end-inspiratory and end-expiratory volumetric CT acquisitions were obtained in the supine position through the entire lungs. The irradiation parameters were adjusted conventionally, according to the morphological characteristics of each patient. The other scan parameters were a slice thickness of 0.75 mm, a reconstruction increment of 0.5 mm, a pitch ratio of 0.8 mm, and a collimation thickness of 0.6 mm. The vendor-recommended kernel filter B20s was used for the quantitative analysis. Images were also reconstructed with standard soft tissue (B41s) and lung parenchyma (B90s) kernel filters.

The data were post-processed using a semiautomatic analysis program (Software syngo InSpace4D; Siemens Healthineer) for pulmonary parenchyma analysis, Siemens Medical Solutions, which provides automated lung segmentations and volumetric and densitometric measures. A noise removal filter was applied to the images. The software combines different techniques for semiautomatic segmentation based on thresholds. The program automatically detects the morphological reference points of the rib cage, outlining the trachea, the right lung, and the left lung. The trachea and the bronchi up to the 8th generation were segmented and excluded from the analysis of the lung parenchyma, since they are considered as part of the “respiratory dead space”. A radiologist reviewed the automatic segmentation to evaluate its adequacy and make minor corrections if necessary. After segmentation, the software provides the following measurements: the total lung volume (TLV) in mL and the relative volume percentages of each of the lungs; the mean lung density (MLD) in Hounsfield Units (HU); the low and high attenuation values (LAV and HAV), expressed as a percentage, with thresholds of −950 HU and −500 HU, respectively; the percentage of lung voxels in the range of emphysema (from −1000 to −950 HU); and the bulla index (BI), which is the percentage of areas covered by small, medium, and large bulla [23]. In addition, all the CT scans were evaluated using the modified Bhalla scoring system by an expert chest radiologist blinded to the clinical data and lung attenuation documented findings in HRCT [12,24,25].

### 2.3. Lung Function Test

Spirometry: The European Respiratory Society/American Respiratory Society (ERS/ATS) guidelines were followed to perform spirometry (MasterScreen 4.0, Viasys, Würtzburg, Germany) [26]. The FEV1 and the forced vital capacity (FVC), expressed in milliliters and as percentage of the prediction (% pred.), were collected. Also, the FEV1/FVC ratio was registered.

Body plethysmography: a MasterScreen body plethysmograph (Viasys, Würtzburg, Germacy) was employed, and ERS/ATS recommendations were used to accomplish it [27]. Body plethysmography allowed for the assessment of the residual volume (RV), total lung capacity (TLC), intrathoracic gas volume (ITGV), expiratory reserve volume (ERV), and airway resistance (Raw). All the volumes were expressed in milliliters and as % pred. Also, the RV/TLC ratio was calculated to assess air trapping.

Statistical analysis: The quantitative variables were expressed as means with standard deviations. For the categorical variables, we used frequencies and proportions. The categorical comparisons were performed using the chi-squared test, and the continuous data were analyzed using an unpaired Student’s *t*-test. For correlations between the variables, we employed Spearman’s correlation coefficients. If the differences were not normally distributed, we employed Spearman’s correlation, using the Kolmogorov–Smirnov test to determine data normality. Finally, to investigate variables related to airflow limitation, univariate and multiple logistic regression analyses were used. The statistical significance was set at a *p*-value of ≤0.05. The data were analyzed by using SPSS Statistics for Windows, Version 24.0. (IBM Corp., Armonk, NY, USA).

## 3. Results

### 3.1. Study Population

In total, 73 subjects (63% males) were included (31.6 ± 12.3 years). CF was diagnosed at the newborn stage in 39 (53.4%) patients, and 16 (21.9%) were homozygous for F508del. The mean modified Medical Research Council scale (mMRC) dyspnea was 1.1 ± 0.9, and 31.5% referred to a severity of dyspnea ≥ 2. Mild and severe respiratory exacerbations in the last year were present in 56 (76.7%) and 14 (19.2%) patients, respectively. Regarding chronic *Pseudomonas aeruginosa* and methicillin-sensitive Staphylococcus aureus (MSSA) lung infections, they were isolated in the sputum of 30 (41.1%) and 41 (56.2%) subjects, respectively (Table 1).

The lung function data, as well as the radiological data, are described in Table 1 and Table 2, respectively.

### 3.2. Comparison Between Non-Airflow Limitation and Airway Limitation Groups

AL was found in 63% of the population. The AL group referred to a higher dyspnea severity than the non-AL patients (1.1 ± 0.9 vs. 0.7 ± 0.8, respectively, *p* = 0.001). Also, systemic inflammatory biomarkers (CRP, ESR, and fibrinogen) were increased in the AL participants when compared with the non-AL group. Finally, the CF patients with ALs had a higher number of severe respiratory events in the previous year than the non-AL subjects (Table 3).

### 3.3. Comparison of Radiological Data

Both at inspiration and at expiration, the percentages of the lungs at the LAV and the HAV were higher in the AL group. Also, at inspiration, the percentage of lung voxels in the range of emphysema and the bulla index were higher in this group, compared with the non-AL group. At expiration, the AL group had a lower MLD than the non-AL patients. Compared with the non-AL group, in the AL group, the TLV inspiratory–expiratory difference was lower and the MLD inspiratory–expiratory difference was higher (Table 3).

Concerning the Bhalla HRCT scoring system, non-significant differences were observed in the total Bhalla score; however, some categories, namely, the extent of mucous plugging (*p* = 0.030) and air trapping (*p* = 0.012), scored higher in the AL patients with respect to the non-AL group.

### 3.4. Comparison of Lung Function

The FEV1% pred. was lower in the AL participants compared to the other group (53.5 ± 17.1 vs. 92.2 ± 19.2, respectively, *p* < 0.001). Also, the FVC, % pred. was lower in this group; however, no differences in the TLC, % pred. were observed between these groups. The RV, % pred. (*p* < 0.001) and the RV/TLC, % pred. were higher in the AL group with respect to the non-AL patients, and, therefore, more patients in the AL group had air trapping in comparison with the non-AL group (97.4% vs. 66.7%, *p* = 0.003) (Table 3).

### 3.5. Correlations Between Radiological Data and Lung Function

The FEV1 and the FEV1/FVC were significantly negatively correlated with the inspiratory LAV, HAV, range of emphysema, and bulla index. At expiration, the FEV1 and the FEV1/FVC were significant positively correlated with the MLD and negatively correlated with the LAV. The inspiratory–expiratory difference in the MLD negatively correlated with the FEV1 (r = −0.460, *p* < 0.001) and the FEV1/FVC (r = −0.480, *p* < 0.001) (Table 4).

At inspiration, the MLD correlated with the ITGV (r= −0.395, *p* = 0.003). At expiration, the MLD had a negative correlation with the RV (r = −0.397, *p* = 0.007), ITGV (−0.500, *p* = 0.001), and RV/TLC (r = −0.363, *p* = 0.014) (Table 4).

### 3.6. Regression Logistic Model

The variables that were found significant in the single logistic regression model were entered into a multivariate model, adjusted for age, sex, and chronic bronchial infection due to *P. aeruginosa*. The MLDi-e (OR 1.034, 95% CI 1.011–1.058, *p* = 0.003) and the HAVi (5.076, 95% CI 1.147–22.456, *p* = 0.032) were found to be independently associated with airflow limitation (Table 5).

## 4. Discussion

The main result of this cross-sectional study is that when we assessed the lung parenchymal attenuation of CF adults by CT, the inspiratory–expiratory variation in the MLD and the HAV on inspiration were associated with airflow obstruction. On the other hand, the bulla index correlated with airflow obstruction, as well as air trapping.

Although many studies have addressed the ability of CT to accurately assess the attributes related to CF lung disease [6,7,8,9,10,11,12,13], as far as we know, the correlation of the attenuation of lung parenchyma and lung function had not been investigated in CF adult patients yet. In this cohort, the variation in the MLD between inspiration and expiration, a standard parameter in the quantitative analysis of the lung, and the inspiratory HAV, a parameter that represents the percentage of voxels above a user-defined attenuation threshold, were correlated with AL. This is an important outcome because airflow obstruction is related to the quality of life and exacerbations, and it is a strong predictor of survival in CF [28]. This finding is supported by various studies on COPD, which have also shown a significant association between the variation in MLD on inspiration and expiration with the presence of AL. Studies have demonstrated that high-resolution computed tomography and pulmonary function tests are common tools that are used to assess the severity of emphysema and small airway dysfunction in smokers. In particular, the expiration/inspiration (E/I) ratio has been associated with hyperinflation and airway obstruction, reflecting morphological changes in lung parenchyma that are relevant in both COPD and cystic fibrosis [29,30]. In addition, in CF, the mucus plugging, bronchiectasis, and wall thickening are responsible for an increase in the HAV, and, at the same time, they are the main causes of airflow obstruction in CF, which can explain our results.

On the other hand, the bulla index is an objective and reliable parameter that serves to quantify, in COPD, the destruction of the parenchyma in addition to determining its location [14]. While in CF there is usually no emphysema, hyperinflation is frequent, especially in those with more severe airflow obstructions. These areas with air trapping and hyperinflation might show less density, as occurs with emphysema. In addition, it is known that the bulla index in COPD is less influenced than other parameters by the presence of coexisting diseases, such as pulmonary fibrosis [23]. Therefore, it is possible that in CF, the bulla index is also less affected by areas with bronchiectasis, mucus plugging, or wall thickening.

The measurement of lung density by automated CT scanning has been used in some studies with children with CF with different objectives. As an example, several investigators showed significant correlations between bronchial dilatation, identified via CT and PFTs, in children with CF. However, they used semiautomated methods, which required readers to measure the airway dimensions [31,32,33]. It has been also used to evaluate responses to treatments. As example, Robinson et al. [34] demonstrated that treatment with dornase alfa in CF subjects improves air trapping. De Lavernhe et al. [21] also showed that automatically calculated indices that quantify both the degree of asymmetry and the lung density sharpness of lung CTs were correlated with both the PFT and the Bhalla score in CF children. However, as far as we are concerned, there is no study which has studied the quantification of lung attenuation in CF adults.

The main weakness of the indices obtained in the quantification of lung attenuation is that they do not provide any information on the lung architectural alterations, such as bronchiectasis, which historically have been considered a factor of severity and a prognosis marker in CF. However, while these indices are not a diagnostic tool, the appropriate integration of automated computer analyses of lung parenchyma attenuation and the functional information provided by the PFTs will improve our ability to phenotype in order to provide better care, and they could be considered as structural end points in interventional studies in CF patients.

On the other hand, the classical parameters of PFTs, such as FVC, FEV1, and FEV1/FVC, had the greatest impact in the follow-up and prognosis of adults with CF [28]. Notwithstanding, it is known that these factors are insensitive to early changes compared to the detailed severity findings in HRCT [6,7,8,9,10,11]. Therefore, several radiologic scoring systems have been proposed for monitoring and quantifying structural pulmonary changes in the CT of CF patients [21]. In fact, they are valuable tools during the patient follow-up because they provide a considerable amount of morphological information that is essential to understanding the natural progression of this disease. Moreover, it is known that they can anticipate the damage revealed by PFTs that could change diagnostic or therapeutic attitudes with the patient [9,10]. While there is not a formal recommendation about which is the best radiologic scoring system, the most well known include the modified Bhalla score, the first scoring system described [6,7,8,10,24], and the Brody II system [8,19,35]. But in addition to the complexity of the Brody II score, they are reader dependent, time consuming, and limited by the number of expert readers available, which makes them difficult to use in daily clinical practice. Thus, while these expert visual scores offer more features of CF lung disease, automated computer analysis can offer important complementary information without the need for specially trained readers, avoids observer bias and variability, offers better standardization, and is a more sensitive technique for detecting subtle variations [18]. Technology, particularly artificial intelligence, is playing an increasingly important role in medicine and has the potential to enhance the analysis of CT features in adult cystic fibrosis patients. In future studies, we aim to incorporate AI-assisted techniques, such as the automatic segmentation of mucus plugging in CT scans, to improve our understanding of lung pathology and its correlation with pulmonary function.

The main strength of this study is the large amount of available information, both functional and morphological. Although this is a cross-sectional study, the correlation found between the lung attenuation parameters and the PFTs opens an opportunity to extend the research in multicentric and prospective studies. It would be also relevant to compare the results of lung parenchyma attenuation with the Bhalla score. Another strength is that we only included adults, because most of the published data about radiological scoring systems were obtained from both adults and children or only children’s cohorts [18,36]. Considering that CF survival is increasing, studies in adults are important.

This study has several limitations. First, it was performed in a single medical center with a limited cohort of patients and, consequently, the findings should be extrapolated with caution to a larger population. Second, since the clinical data were collected retrospectively from the medical records, some data could not have been available. We strongly believe that a prospective study confirming these results would be very interesting, in order to identify the evolution of these radiological parameters and their correlation with clinical and functional data.

## 5. Conclusions

In conclusion, our results support that the automated quantification of lung parenchymal attenuation is a valuable complementary tool in the management of CF. It provides indices such as the variation between the MLD on inspiration and expiration and the HAV, which are related to airflow obstruction, and the bulla index, which is correlated with bronchial obstruction and air trapping. The logistic regression results indicate that both the MLD on expiration and the HAV on inspiration are significant predictors of respiratory function in cystic fibrosis patients, independent of demographic and clinical factors such as age, sex, and the presence of chronic bronchial infection due to *Pseudomonas aeruginosa*. The analysis shows that the MLD measured during expiration is associated with poorer respiratory function. This suggests that as the MLD decreases (indicating more air trapping or structural changes in the lungs), the likelihood of experiencing a worse lung function increases. Similarly, the HAV measured during inspiration is also linked to worse respiratory outcomes. A higher attenuation value during inspiration may indicate the presence of denser lung tissue or areas of consolidation, which can be detrimental to lung function. This highlights the importance of radiological assessments in evaluating disease severity and guiding management strategies in this population. Inflammatory markers, such as C-reactive protein, are significantly elevated in patients with poorer pulmonary function and patients with a higher number of respiratory exacerbations in the previous year. This suggests that as lung function declines, there is a corresponding increase in systemic inflammation, which may contribute to the progression of respiratory disease in CF.

These findings also point to the need for further research to explore the underlying mechanisms linking radiological changes to respiratory outcomes in CF. Understanding how these variables interact with other clinical factors could enhance the development of targeted therapies aimed at improving lung function and reducing exacerbations.

## Figures and Tables

**Table 1:** Characteristics of patients.

Variable		*n* = 73
Clinical Characteristics		
Males, %		46 (63)
Age, years		31.6 ± 12.3
BMI, kg/m^2^		21.9 ± 2.9
Genotype, %		
	F508del homozygous	16 (21.9)
	F508del heterozygous	34 (46.6)
	^1^ Other	23 (31.5)
Dyspnea mMRC		1.1 ± 0.9
Dyspnea ≥ 2 mMRC, %		23 (31.5)
Previous respiratory CF complications, %		
	Atelectasis	9 (12.3)
	Haemoptysis	11 (15.1)
	^2^ Other	15 (20.5)
Previous or concomitant CF systemic complications, %		
	Exocrine pancreatic insufficiency	49 (67.1)
	CFRD	18 (24.7)
	Liver disease	25 (34.2)
	^3^ Other	13 (17.8)
Number of mild respiratory exacerbations in the previous year		1.9 ± 1.5
Number of severe respiratory exacerbations in the previous year		0.3 ± 0.7
Mild respiratory exacerbations in the previous year, %		56 (76.7)
Severe respiratory exacerbations in the previous year, %		14 (19.2)
Chronic bronchial infection, %		
	*Pseudomonas aeruginosa*	30 (41.1)
	MSSA	41 (56.2)
	MRSA	9 (12.3)
	Fungal chronic infection	14 (19.2)
	Nontuberculous mycobacteria	6 (8.2)
Biochemical analysis		
CRP, mg/L		10.7 ± 11
ESR, mm/h		15.3 ± 11.7
Fibrinogen, mg/dL		442 ± 113.3
Prealbumin, mg/dL		23.1 ± 5.1
Albumin, g/dL		4.3 ± 0.3
Proteins, g/dL		7.6 ± 0.5
Pulmonary function tests		
Spirometry		
	FEV_1_, mL	2454.5 ± 1044.4
	FEV_1_, % pred.	67.8 ± 25.9
	FVC, mL	3712.3 ± 1209.5
	FVC % pred.	86.7 ± 21.5
	FEV_1_/FVC	64.7 ± 12.7
Body plethysmography		
	RV, mL	2497.4 ± 749.1
	RV, % pred.	159.9 ± 46.3
	TLC, mL	6214.5 ± 1225.2
	TLC, % pred.	105.3 ± 13.6
	ITGV, mL	3721.1 ± 905.4
	ITGV, % pred.	122.7 ± 21.3
	RV/TLC, % pred.	145.5 ± 46.3
	R_aw_, % pred.	184.1 ± 81.2
Others		
	^4^ Airflow limitation, %	46 (63)
	^5^ Severe airflow limitation, %	18 (24.7)
	^6^ Air trapping, %	50 (87.7)

Data are presented as mean ± SD or number (percentage). BMI = Body mass index; CF = cystic fibrosis; CFRD = cystic fibrosis-Related Diabetes; CRP = C-reactive protein; ESR = erythrocyte sedimentation rate; FEV_1_ = forced expiratory volume in one second; FVC = forced vital capacity; ITGV = intrathoracic gas volume; mMRC = modified Medical Research Council scale; MRSA = methicillin-resistant *Staphylococcus aureus*; MSSA = methicillin-sensitive *Staphylococcus aureus*; R_aw_ = airway resistance; RV = residual volume; TLC = total lung capacity. ^1^ Including G542/F334W, G542/R1162X, G542X/1812-1G>A, G542X/ASP443Tyr, G542X/R1162X, G542X/R334W, G542X/V232D, G673X/2789+5G>A, G85E/G85E, I507del/711+1G>T, I507Del/R334W, I507Del/R553X, L206W/5T-11TG, N1303k/5T, p.Leu365Pro/p.Arg1066Cys, c.489+1G>T/p.Val1008Asp, Q1313X/D1152H, R334W/R1066C, W1282stop/I507del, and UNK. (UNK: unknow). ^2^ Including allergic bronchopulmonary aspergillosis, community acquired pneumonia, mycetoma, pulmonary embolism, pulmonary hypertension, and pulmonary tuberculosis. ^3^ Including azoospermia, bile duct disease, distal intestinal obstruction, gastroesophageal reflux, meconium ileus, obstructive uropathy, osteopenia, and pancreatitis. ^4^ Airflow limitation was defined as FEV_1_/FVC < 0.7. ^5^ A severe airflow obstruction was defined as FEV_1_/FVC < 0.7 and FEV_1_ < 50% pred. ^6^ Air trapping was defined as RV/TLC > 120% pred.

**Table 2:** Computed tomography scan characteristics.

Computed Tomography Scan Characteristics		
Inspiratory		
	Total lung volume, mL	5470.8 ± 1275.4
	Mean lung density, HU	−821.7 ± 33.5
	^1^ Low attenuation value, %	4.7 ± 4
	^2^ High attenuation value, %	1.9 ± 0.8
Expiratory		
	Total lung volume, mL	3683.6 ± 1044.3
	Mean lung density, HU	−733.4 ± 65.8
	^1^ Low attenuation value, %	1.7 ± 1.9
	^2^ High attenuation value, %	3.1 ± 1.4
	^3^ Range of emphysema, %	2.5 ± 3.2
	Bulla index	0.9 ± 1.3
Inspiratory—Expiratory difference		
	Total lung volume, mL	1918.6 ± 881.9
	Mean lung density, HU	−92.6 ± 63.7
	^1^ Low attenuation value, %	3.9 ± 3.4
	^2^ High attenuation value, %	−1.1 ± 1.1
Bhalla high-resolution computed tomography scoring system		14.6 ± 2.7

Data are presented as mean ± SD. HU = Hounsfield units. ^1^ A low attenuation value was defined as a percentage with a threshold of −950 HU. ^2^ A high attenuation value was defined as a percentage with a threshold of −500 HU. ^3^ The range of emphysema was defined as a percentage of lung voxels in the range −1000 to −950 HU.

**Table 3:** Comparison of patients categorized by airflow limitation.

Variable		Non-Airflow Limitation (*n* = 27)	Airflow Limitation (*n* = 46)	*p*
Demographic data				
Males, %		18 (66.7)	28 (60.9)	0.620
Age, years		30.4 ± 14.9	32.2 ± 10.7	0.084
BMI, kg/m^2^		22.5 ± 3.3	21.5 ± 2.7	0.201
Genotype, %				0.063
	F508del homozygous	2 (7.4)	14 (30.4)	
	F508del heterozygous	14 (51.9)	20 (43.5)	
	^1^ Other	11 (40.7)	12 (26.1)	
Dyspnea mMRC		0.7 ± 0.8	1.1 ± 0.9	0.001
Dyspnea ≥ 2 mMRC, %		5 (18.5)	18 (39.1)	0.067
CRP, mg/L		5.9 ± 7.7	13.4 ± 11.8	<0.001
CRP > 5 mg/L, %		9 (36)	33 (75)	0.001
ESR, mm/h		10.3 ± 6.9	17.9 ± 12.9	0.023
Fibrinogen, mg/dL		406.4 ± 95.1	461.8 ± 118.6	0.041
Number of mild respiratory exacerbations in the previous year		1.7 ± 1.6	1.9 ± 1.5	0.532
Number of severe respiratory exacerbations in the previous year		0.1 ± 0.3	0.4 ± 0.8	0.045
Chronic bronchial infection, %				
	*Pseudomonas aeruginosa*, %	8 (29.6)	22 (47.8)	0.127
	MSSA, %	16 (59.3)	25 (54.3)	0.683
	MRSA, %	1 (3.7)	8 (17.4)	0.141
	Fungal chronic infection	2 (7.4)	12 (26.1)	0.050
	Nontuberculous mycobacteria	2 (7.4)	4 (8.7)	1
Computed tomography scan characteristics				
Inspiratory				
	Total lung volume, mL	5278.6 ± 1276.2	5583.6 ± 1275.3	0.323
	Mean lung density, HU	−823.9 ± 29.7	−820.3 ± 35.8	0.859
	^2^ Low attenuation value, %	2.9 ± 3.2	5.7 ± 4.1	0.002
	^3^ High attenuation value, %	1.6 ± 0.5	2.2 ± 0.9	0.002
	^4^ Range of emphysema, %	1.1 ± 1.7	3.2 ± 3.6	<0.001
	Bulla index	0.3 ± 0.4	1.3 ± 1.5	<0.001
Expiratory				
	Total lung volume, mL	3300.8 ± 1138.9	3874.9 ± 953.1	0.033
	Mean lung density, HU	−691.4 ± 82.9	−754.5 ± 43.1	0.002
	^2^ Low attenuation value, %	0.7 ± 0.7	2.2 ± 2.1	<0.001
	^3^ High attenuation value, %	2.9 ± 1.2	3.3 ± 1.6	0.419
Inspiratory—Expiratory difference				
	Total lung volume, mL	2300.8 ± 725.7	1727.5 ± 899.6	0.013
	Mean lung density, HU	−141.1 ± 77.6	−68.4 ± 37.6	<0.001
	^2^ Low attenuation value, %	3.4 ± 3.3	4.2 ± 3.4	0.399
	^3^ High attenuation value, %	−1.3 ± 1	−1 ± 1.1	0.183
Total Bhalla score		15.1 ± 2.6	14.3 ± 2.7	0.277
Pulmonary function tests				
Spirometry				
	FEV_1_, mL	3345.9 ± 837.7	1931.3 ± 763.6	<0.001
	FEV_1_, % pred.	92.2 ± 19.2	53.5 ± 17.1	<0.001
	FVC, mL	4322.2 ± 1125.4	3354.3 ± 1120.2	0.001
	FVC, % pred.	99.8 ± 17.8	79.1 ± 19.8	<0.001
	FEV_1_/FVC	78.2 ± 6.7	56.8 ± 7.9	<0.001
Body plethysmography				
	RV, mL	1975.1 ± 554.8	2738.9 ± 706.8	<0.001
	RV, % pred.	126.1 ± 34.3	175.4 ± 42.8	<0.001
	TLC, mL	6182.7 ± 1189	6229.2 ± 1256.5	0.877
	TLC, % pred.	100. ± 8.2	107.2 ± 15.2	0.101
	ITGV, mL	3468.2 ± 960.6	3834.1 ± 868.8	0.089
	ITGV, % pred.	109.4 ± 18.2	128.7 ± 20	0.002
	RV/TLC, % pred.	114.7 ± 37.6	159.7 ± 43.3	<0.001
	R_aw_, % pred.	183.9 ± 103.6	184.1 ± 70.2	0.542
Others				
	^5^ Air trapping, %	12 (66.7)	38 (97.4)	0.003

Comparisons between groups by Mann–Whitney U test and chi-squared test. BMI = Body mass index; CF = cystic fibrosis; CRP = C-reactive protein; ESR = erythrocyte sedimentation rate; FEV_1_ = forced expiratory volume in one second; FVC = forced vital capacity; HU = Hounsfield units; ITGV = intrathoracic gas volume; mMRC = modified Medical Research Council scale; MRSA = methicillin-resistant *Staphylococcus aureus*; MSSA = methicillin-sensitive *Staphylococcus aureus.* R_aw_ = airway resistance; RV = residual volume; TLC = total lung capacity. ^1^ Including G542/F334W, G542/R1162X, G542X/1812-1G>A, G542X/ASP443Tyr, G542X/R1162X, G542X/R334W, G542X/V232D, G673X/2789+5G>A, G85E/G85E, I507del/711+1G>T, I507Del/R334W, I507Del/R553X, L206W/5T-11TG, N1303k/5T, p.Leu365Pro/p.Arg1066Cys, c.489+1G>T/p.Val1008Asp, Q1313X/D1152H, R334W/R1066C, W1282stop/I507del, and UNK. (UNK: unknown). ^2^ A low attenuation value was defined as a percentage with a threshold of −950 HU. ^3^ A high attenuation value was defined as a percentage with a threshold of −500 HU. ^4^ The range of emphysema was defined as a percentage of lung voxels in the range −1000 to −950 HU. ^5^ Air trapping was defined as RV/TLC > 120% pred.

**Table 4:** Correlations between lung function tests and computed tomography scans results.

Variable		FEV_1_ (% Pred)	*p*	FEV_1_/FVC	*p*	RV (% Pred)	*p*	TLC (% Pred)	*p*	ITGV (% Pred)	*p*	RV/TLC	*p*
Inspiratory													
	TLV, mL	−0.040	0.737	−0.259	0.027	0.168	0.213	0.105	0.439	0.432	0.001	0.034	0.799
	MLD, HU	−0.128	0.280	0.080	0.503	−0.051	0.706	−0.181	0.179	−0.395	0.003	0.148	0.271
	LAV, %	−0.351	0.002	−0.448	<0.001	0.267	0.044	0.164	0.222	0.461	<0.001	0.192	0.153
	HAV, %	−0.415	<0.001	−0.333	0.004	0.097	0.475	−0.046	0.732	−0.131	0.340	0.261	0.050
	Range of emphysema, %	−0.528	<0.001	−0.468	<0.001	0.180	0.241	−0.200	0.193	0.115	0.465	0.331	0.028
	Bulla index	−0.447	<0.001	−0.519	<0.001	0.389	0.003	0.119	0.378	0.286	0.034	0.329	0.012
Expiratory													
	TLV, mL	−0.242	0.077	−0.427	0.001	0.335	0.024	0.098	0.522	0.434	0.003	0.318	0.033
	MLD, HU	0.353	0.009	0.448	0.001	−0.397	0.007	−0.144	0.346	−0.500	0.001	−0.363	0.014
	LAV, %	−0.516	<0.001	−0.518	<0.001	0.382	0.010	0.283	0.060	0.136	0.378	0.419	0.004
	HAV, %	−0.222	0.107	−0.179	0.194	−0.034	0.822	0.068	0.657	−0.121	0.434	−0.003	0.982
Inspiratory—Expiratory difference													
	TLV mL	0.295	0.030	0.0236	0.085	−0.023	0.883	0.023	0.881	0.296	0.051	−0.134	0.379
	MLD, HU	−0.460	<0.001	−0.480	<0.001	0.378	0.010	0.084	0.582	0.288	0.058	0.412	0.005
	LAV, %	−0.084	0.548	−0.172	0.215	0.083	0.586	0.028	0.856	0.431	0.004	−0.014	0.928
	HAV, %	−0.035	0.803	−0.055	0.690	0.116	0.447	0.098	0.523	0.068	0.663	0.137	0.368

Correlations between variables by employed Spearman’s correlation test. FEV_1_ = forced expiratory volume in one second; FVC = forced vital capacity; HAV = high attenuation value; HU = Hounsfield units; ITGV = intrathoracic gas volume; LAV = low attenuation value; MLD = mean lung density; RV = residual volume; TLC = total lung capacity; TLV = total lung volume. A low attenuation value was defined as a percentage with a threshold of −950 HU. A high attenuation value was defined as a percentage with a threshold −500 HU. The range of emphysema was defined as a percentage of lung voxels in the range −1000 to −950 HU.

**Table 5:** Variables associated with airflow limitation in patients diagnosed with cystic fibrosis.

Variable	B	Standard Error	OR	95% CI	*p*
MLDi-e	0.034	0.011	1.034	1.011–1.058	0.003
HAVi	1.624	0.759	5.076	1.147–22.456	0.032
Constant	0.962	1.605	-	-	-

Multivariate model adjusted for age, sex, and chronic bronchial infection due to *Pseudomonas aeruginosa*. B = beta coefficient; CI = confidence interval; MLDi-e = mean lung density in inspiration-expiration difference; HAVi = high attenuation value in inspiration; OR = odds ratio.

## Data Availability

The original contributions presented in the study are included in the article; further inquiries can be directed to the corresponding author.
